# Case Report: A case of atypical persistent proatlantal intersegmental artery

**DOI:** 10.3389/fsurg.2026.1820020

**Published:** 2026-06-08

**Authors:** Xiaobin Sun, Dajiang Xie, Xuchen Qi

**Affiliations:** Department of Neurosurgery, Sir Run Run Shaw Hospital, School of Medicine, Zhejiang University, Hangzhou, China

**Keywords:** anatomical variation, external carotid artery, occipital artery, persistent proatlantal intersegmental artery, vertebral artery agenesis

## Abstract

The persistent proatlantal intersegmental artery (PPIA) is a rare embryonic anastomosis between the carotid and vertebrobasilar systems. We report an unusual case of PPIA in a 67-year-old male presenting with recurrent dizziness, which cannot be classified into the classic Lasjaunias types. Imaging revealed absence of the left vertebral artery, a right vertebral artery V4 segment aneurysm, and an anomalous artery originating from the left external carotid artery that gave off the occipital artery and ascended directly through the occipitoatlantal space into the foramen magnum without entering any cervical transverse foramen, ultimately joining the right vertebral artery to form the basilar artery. This variant exhibits a type II origin with a type I course, representing a functional composite artery formed by persistent remnants of both type I and type II proatlantal intersegmental artery (PIA) in the setting of ipsilateral vertebral artery agenesis. Our findings support the embryological concept that the occipital artery derives from PIA components and expand the morphological spectrum of persistent proatlantal arteries. Recognition of such “mixed-type” PPIA is clinically crucial as it may serve as the dominant or sole posterior circulation supply and constitutes a potential dangerous anastomosis; preoperative identification via CTA or MRA is essential for surgical and interventional planning to avoid catastrophic complications.

## 1 Introduction

The persistent proatlantal intersegmental artery (PPIA) is a rare remnant of embryonic anastomotic vessels connecting the carotid and vertebrobasilar systems, exhibiting a low incidence among persistent carotid-vertebrobasilar anastomoses. According to the classic Lasjaunias classification (1), PPIA is divided into two types: Type I originates from the internal carotid artery or common carotid artery, courses through the preatlantal space without entering the transverse foramen, and joins the ipsilateral vertebral artery at the V3 segment. Type II originates from the external carotid artery, runs posterosuperiorly to enter the C1 transverse foramen, traverses the atlantoaxial space, and merges with the vertebral artery before entering the skull via the foramen magnum. The vascular variation in the present patient is highly unusual and cannot be fully classified into either type. Its imaging characteristics and hemodynamic features provide a unique perspective for understanding the embryonic development and variations of craniocervical vessels.

## 2 Case report

A 67-year-old male presented with recurrent paroxysmal dizziness over a period of 5 months. His medical history included hypertension, smoking and alcohol abuse. Neurological examination revealed no significant abnormalities. Computed tomography angiography (CTA) demonstrated a right vertebral artery aneurysm, absence of the left vertebral artery, and a vessel originating from the left external carotid artery. After giving off the occipital artery, this vessel ascended intracranially through the foramen magnum to join the right vertebral artery, forming the basilar artery (Figure 1). Digital subtraction angiography (DSA) confirmed a right vertebral artery V4 segment aneurysm and a vessel arising from the left external carotid artery supplying the posterior circulation, with no other carotid-vertebrobasilar anastomoses visualized. Three-dimensional reconstruction showed that the artery originated from the external carotid artery and gave off the occipital artery. It then traversed the occipitoatlantal space, entered the skull via the foramen magnum, and did not enter any cervical transverse foramen during its course (Figure 2). The patient was successfully treated with flow diverter implantation in the right vertebral artery and was discharged.

**Figure 1:**
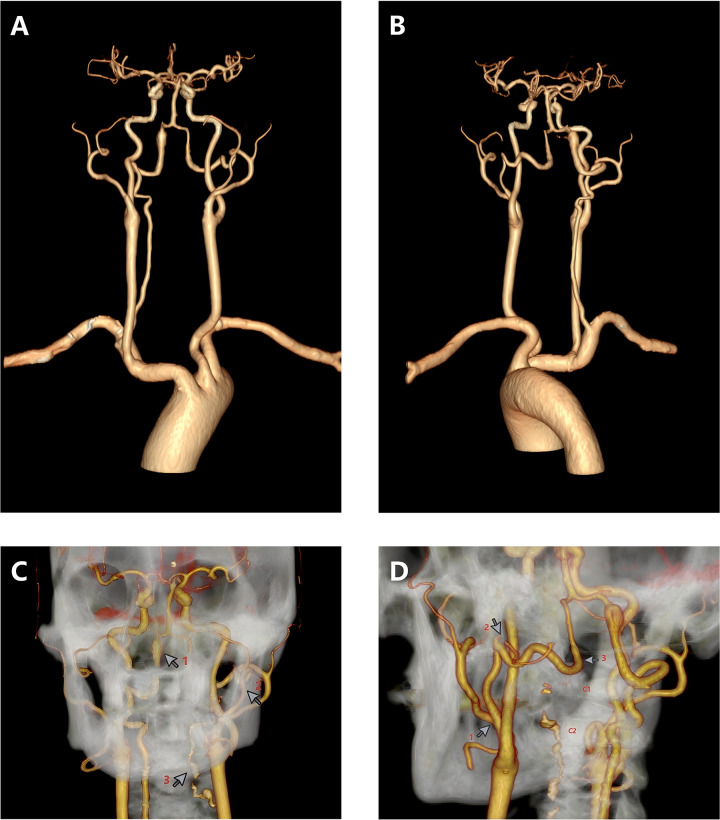
**(A,B)** the computed tomography angiography (CTA) covering the aortic arch to the intracranial region. **(C)** 3D CTA reconstruction shows an aneurysm of the right vertebral artery (1). An anomalous vessel originating from the left external carotid artery gives off the occipital artery and then courses intracranially to join the right vertebral artery, forming the basilar artery (2). The left vertebral artery is present only as a remnant (3). **(D)** Posterolateral 3D CTA reconstruction shows an anomalous vessel originating from the left external carotid artery (1). After giving off the occipital artery (2), it passes through the atlanto-occipital space and enters the cranium via the foramen magnum (3) to join the right vertebral artery in forming the basilar artery.

**Figure 2:**
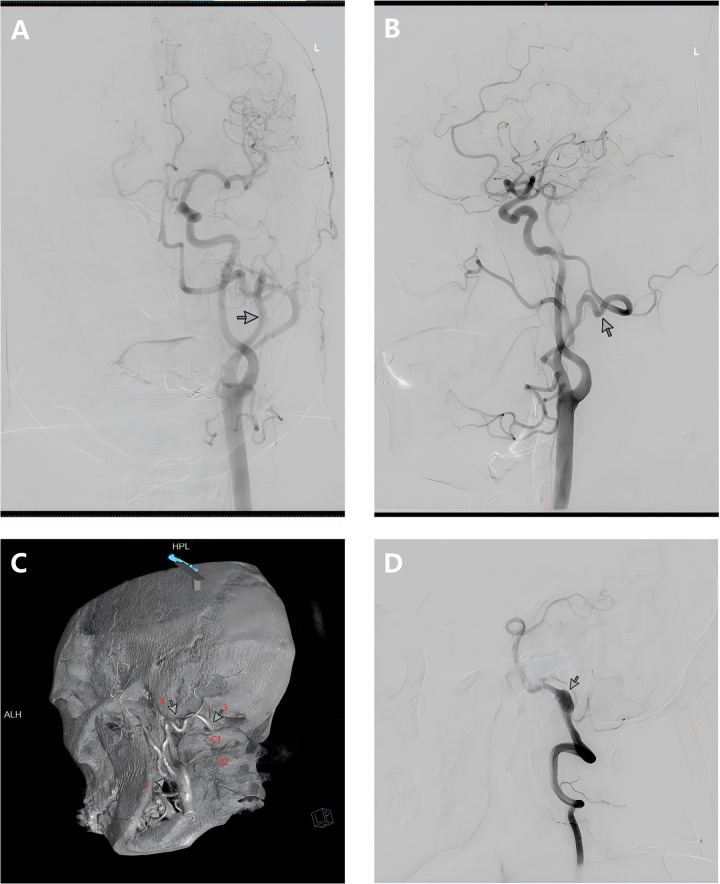
**(A,B)** anteroposterior and left lateral projections of left common carotid angiography demonstrate a vessel originating from the left external carotid artery that supplies the posterior circulation. No other carotid-vertebrobasilar anastomoses are observed. **(C)** 3D reconstruction shows the artery originating from the external carotid artery (1). After giving off the occipital artery (2), it passes through the atlanto-occipital space and enters the cranium via the foramen magnum (3). The vessel does not traverse any cervical transverse foramen along its course. **(D)** Left lateral projection of right vertebral artery angiography shows an aneurysm of the right vertebral artery.

## 3 Discussion

According to Padget's classic studies (2), during the 4–5 mm stage of the human embryo (approximately day 30), the posterior circulation is supplied by two parallel neural arteries, which receive blood flow through temporary carotid-vertebrobasilar anastomoses, including the trigeminal, otic, hypoglossal, and proatlantal arteries. During the 7–12 mm stage (approximately day 33), the vertebral artery forms gradually through a longitudinal anastomotic network connecting the cervical intersegmental arteries. In this network, the first cervical intersegmental artery corresponds to proatlantal intersegmental artery (PIA) Type I, and the second corresponds to PIA Type II.Normally, these temporary anastomotic vessels regress with the development of the posterior communicating arteries, and their remnants contribute to the formation of extracranial vessels such as the occipital artery.

The embryological model proposed by Lasjaunias et al. suggests that the occipital artery evolves from remnants of PIA Types I and II: the horizontal segment and distal ascending segment of the occipital artery derive from Type I PIA, while the proximal ascending segment and C2 muscular branch derive from Type II PIA (3). In the present case, the artery originated from the external carotid artery (consistent with Type II origin), did not enter any cervical transverse foramen during its course (consistent with Type I course), gave off the occipital artery, and entered the skull via the foramen magnum, accompanying ipsilateral vertebral artery hypoplasia, ultimately joining the basilar artery.The unique formation of this PPIA may have resulted from hypoplasia of the ipsilateral vertebral artery during early embryonic development, leading to insufficient posterior circulation supply. It is hypothesized that, the distal segment of a Type I PIA, which would normally regress completely or persist only as a small anastomotic branch between the occipital and vertebral arteries, may have been retained and dilated. The proximal segment of a Type II PIA, which normally develops into the proximal occipital artery, may have assumed the function of connecting the external carotid artery to the posterior circulation, becoming the main conduit supplying the posterior circulation. Similar phenomena have been reported in other cases (4, 5), suggesting that such an “occipital artery-type” or “mixed-type” PPIA may not be an isolated finding but represents a subtype within the morphological spectrum of PPIA.

PPIA is typically asymptomatic, but its presence must be considered in clinical practice, particularly in the diagnosis and treatment of craniocervical vascular lesions. Reports indicate that PPIA can coexist with ipsilateral vertebral artery hypoplasia and contralateral vertebral artery occlusion, making the posterior circulation entirely dependent on this anomalous artery (6). Under these circumstances, PPIA can represent a veritable “lifeline”. During ipsilateral cervical or skull base surgeries (e.g., carotid endarterectomy, cervical mass resection, anterior cervical approach surgery) or endovascular interventions (such as carotid stenting or embolization procedures), accurate preoperative identification of the PPIA via CTA or MRA is essential to avoid inadvertent intraoperative occlusion or injury. Grego et al. reported a strategy of using a balloon shunt to protect the PPIA during carotid endarterectomy, preserving both anterior and posterior circulation blood flow, which offers a valuable reference (7).

Furthermore, PPIA directly connects the external carotid artery to the basilar artery, creating a dangerous channel. Bahsi et al. reported a case of “top of the basilar” syndrome resulting from carotid artery plaque embolization via a Type I PPIA (8). Therefore, if vulnerable plaques exist in the external carotid artery system or during liquid embolic agent therapy, emboli or embolic material could inadvertently enter the posterior circulation through this channel, causing iatrogenic stroke.

Additionally, PPIA can provide a pathway for posterior circulation interventions. Elder et al. reported endovascular treatment of a basilar tip aneurysm via a persistent PPIA, detailing principles for establishing access and catheter selection when PPIA is the sole conduit to the posterior circulation (9).The therapeutic implications of this anatomy are further illustrated by a recent report in which a type II PPIA dissection causing an embolic shower was successfully managed with pipeline embolization device-assisted angioplasty (10). This highlights not only the potential for the PPIA to be the source of embolic pathology but also that it can serve as a conduit for advanced endovascular treatment.

## 4 Conclusion

This case reports a rare PPIA variation that cannot be simply classified. Its essence is a functional composite feeding artery, developed from remnants of both embryonic PIA types (Type I and Type II) against the background of ipsilateral vertebral artery absence. This case enriches our understanding of the imaging spectrum of PPIA and deepens our comprehension of craniocervical vascular development. In the diagnosis and treatment of craniocervical vascular lesions, recognizing such variant vessels is of paramount importance for formulating safe surgical or interventional strategies and avoiding severe complications.

## Data Availability

The original contributions presented in the study are included in the article/Supplementary Material, further inquiries can be directed to the corresponding author.
